# Balancing Gender Bias in Job Advertisements With Text-Level Bias Mitigation

**DOI:** 10.3389/fdata.2022.805713

**Published:** 2022-02-18

**Authors:** Shenggang Hu, Jabir Alshehabi Al-Ani, Karen D. Hughes, Nicole Denier, Alla Konnikov, Lei Ding, Jinhan Xie, Yang Hu, Monideepa Tarafdar, Bei Jiang, Linglong Kong, Hongsheng Dai

**Affiliations:** ^1^Department of Mathematical Sciences, University of Essex, Colchester, United Kingdom; ^2^Department of Strategy, Entrepreneurship and Management, and Sociology, University of Alberta, Edmonton, AB, Canada; ^3^Department of Sociology, University of Alberta, Edmonton, AB, Canada; ^4^Department of Mathematical and Statistical Sciences, University of Alberta, Edmonton, AB, Canada; ^5^Department of Sociology, Lancaster University, Lancaster, United Kingdom; ^6^Isenberg School of Management, University of Massachusetts, Amherst, MA, United States

**Keywords:** bias evaluation, bias mitigation, constrained sampling, gender bias, importance sampling

## Abstract

Despite progress toward gender equality in the labor market over the past few decades, gender segregation in labor force composition and labor market outcomes persists. Evidence has shown that job advertisements may express gender preferences, which may selectively attract potential job candidates to apply for a given post and thus reinforce gendered labor force composition and outcomes. Removing gender-explicit words from job advertisements does not fully solve the problem as certain implicit traits are more closely associated with men, such as *ambitiousness*, while others are more closely associated with women, such as *considerateness*. However, it is not always possible to find neutral alternatives for these traits, making it hard to search for candidates with desired characteristics without entailing gender discrimination. Existing algorithms mainly focus on the detection of the presence of gender biases in job advertisements without providing a solution to how the text should be (re)worded. To address this problem, we propose an algorithm that evaluates gender bias in the input text and provides guidance on how the text should be debiased by offering alternative wording that is closely related to the original input. Our proposed method promises broad application in the human resources process, ranging from the development of job advertisements to algorithm-assisted screening of job applications.

## 1. Introduction

Despite progress toward gender equality at work in recent years, gender segregation in the composition of the labor force remains and clear gender differences in labor market outcomes persist (Bertrand, 2020; England et al., 2020). The hiring process is a critical point in addressing gender inequality. It is well established that gender signaling in job advertising plays an important role in shaping the gender composition of the labor market and workforce across different industries and occupations. The difference in how a job post is perceived by male and female applicants^1^ may stem from different causes, including gender stereotypes (Glick and Fiske, 1996), differences in the everyday language of men and women (Pennebaker et al., 2003), and different linguistic styles (Lakoff, 1973; Carli, 1990). Whatever the underlying cause, gender-definite words and attribute words that seem gender-neutral are shown to contribute to signaling gender preference in job posts (Bem and Bem, 1973; Born and Taris, 2010). Job posts with gender preference are perceived differently by male and female applicants and can discourage potential applicants of the opposite gender from applying even if they are qualified.

Bias detection and evaluation in job text are usually done by targeting specific words that are more commonly associated with a specific gender, e.g., *ambitious* is usually considered masculine and *considerate* is usually considered feminine even though both words can be used to describe people of any gender. Studies such as Gaucher et al. (2011) and Tang et al. (2017) evaluate gender bias by counting target words and computing accumulated weight for words that are classified into feminine and masculine categories. Another approach to bias evaluation relies on a family of natural language processing (NLP) techniques called *word embeddings* such as Word2Vec (Mikolov et al., 2013), GloVe (Pennington et al., 2014), etc. A word embedding model encodes each word in its dictionary into a real vector in high-dimensional space. It is shown that word embeddings are also able to encode information to denote “gender direction” in vectors. For instance, the vector of *he* − *she* points to a similar direction as the vector *father* − *mother*. Thus, cosine similarity can be used to test if a word is biased toward a certain direction of gender (i.e., masculine/feminine) (Caliskan et al., 2017; Garg et al., 2018; Kwak et al., 2021).

Bias mitigation in NLP models has received considerable attention (Bolukbasi et al., 2016; Zhao et al., 2018; Dev and Phillips, 2019; Kaneko and Bollegala, 2019; Wang et al., 2020; Ding et al., 2021). However, the definition of gender-neutral words in the NLP community includes all words that do not explicitly refer to a certain gender. The goal of this research lies in removing gender stereotypes in gender-neutral words perceived by machine learning models and decoupling gender information from semantic information to avoid the incorrect association of attributes to gender due to the presence of gender stereotypes in the training corpus. This procedure allows the models to make predictions free of gender stereotypes. This is different from bias mitigation in the text which requires the model to actively recognize gender bias in words and redesign the wording to reduce the bias perceived by humans.

To the best of our knowledge, there is no off-the-shelf algorithm that can detect and mitigate bias in an input text. The approach closest to our interest may be *paraphrase generation* where the algorithm is designed to paraphrase a piece of text, usually a sentence, by imposing constraints that include and exclude certain words (Swanson et al., 2014; Hokamp and Liu, 2017; Miao et al., 2019). However, existing algorithms do not scale well with the size of the vocabulary constraint and are not able to deal with soft constraints such as using *n* out of *m* words in a given list.

To remedy the above important gaps in existing research, we develop an algorithm that can provide guidance in word composition to express low gender bias. Since certain words in job posting are hard to replace even though they are biased toward a certain gender, when changing the word composition, it is important for the debiased composition to replace as few words as possible. To achieve this goal, we develop a novel method that models both gender bias in words and their word frequencies, and samples a word composition that reduces biases while making few changes to the original wording.

The rest of the paper is organized as follows. First, a more detailed background on bias in the job market and bias evaluation is included in Section 2. Then, in Section 3, we describe the implementation details of our algorithm. The algorithm is applied to a real job text dataset and results are presented in Section 4. Finally, we turn to the discussion in Section 5.

## 2. Related Works

### 2.1. Gender Bias in Job Advertisement

Gender inequality in the labor market is longstanding and well-documented. Although there has been a long-term increase in women's labor force participation over the past few decades, research shows persistent gender segregation across many occupations and industries. Women continue to be underrepresented in senior and managerial positions (Sohrab et al., 2012), are less likely to be promoted and are perceived as less committed to professional careers (Wallace, 2008) and as less suitable to perform tasks in the fields that have been historically male-dominated (Hatmaker, 2013). The hiring process is a significant social encounter, in which employers search for the most “suitable” candidate to fill the position (Kang et al., 2016; Rivera, 2020). Research demonstrates that “suitability” is often defined categorically, is not neutral to bias, and is gendered (McCall, 2005). The wording of job advertisements, in particular, may play a role in generating such gender inequality. For instance, Bem and Bem (1973) and Kuhn et al. (2020) show that job advertisements with explicitly gendered words discourage potential applicants of the opposite gender from applying, even when they are qualified to do so, which in turn reinforces the imbalance. More recent studies (Born and Taris, 2010; Askehave and Zethsen, 2014) have shown that words describing gendered traits and behaviors may also entail gendered responses from potential job applicants. Female students are substantially more attracted to advertisements that contain feminine traits than masculine traits (Born and Taris, 2010). Traits favored in leadership roles are predominately considered to be male-biased, correlating with the gender imbalance in top-management positions (Askehave and Zethsen, 2014). It has been shown that such bias co-exists with the salary gap where, on average, job posts that favor masculine traits offer higher salaries compared with job posts that favor feminine traits (Arceo-Gómez et al., 2020). Research also shows that using gender-neutral terms (e.g., police officer) or masculine/feminine pairs (e.g., policeman/policewoman) can help reduce gender barrier and attract both male and female applicants (Bem and Bem, 1973; Horvath and Sczesny, 2016; Sczesny et al., 2016).

### 2.2. Bias Evaluation at the Text Level

Many studies can be found that collect and identify masculine and feminine words as a measure of gendered wording (Bem and Bem, 1973; Bem, 1974; Gaucher et al., 2011). These word lists are consistent with previous research that examined gender differences in language use (Newman et al., 2008). Given the list of gender-coded words, text-level bias can be quantified by measuring the occurrences of each word in the list. Gaucher et al. (2011) calculated the percentage of masculine and feminine words in the text to produce two separate scores, for male and female biases, respectively, to reveal the fact that job advertisements in male-dominated industries and female-dominated industries exhibit different score pairs. Tang et al. (2017) presents a slightly different approach where they assign weights to each gendered word by their level of gender implications that accumulate over the whole text, with the effects of masculine words and feminine words offsetting each other Tang et al. (2017).

Another technique of bias evaluation relies on the use of word embeddings. Using this technique, we can evaluate the level of bias owing to the fact that gender stereotype bias can be passed on from corpus to the embedding model through training (Bolukbasi et al., 2016). The Word Embedding Association Test (WEAT), proposed by Caliskan et al. (2017), is an analog to the Implicit Association Test (IAT) used in Psychology studies. The purpose of WEAT is to test and quantify that two groups of target words, e.g., male-dominated professions vs. female-dominate professions, are indeed biased toward two groups of attribute words, e.g., {*he*}, {*she*}. A similar strategy is developed in Garg et al. (2018) called Relative Norm Distance (RND) which tests a single group of target words against two groups of attribute words, though the idea is much the same as WEAT. The bias of each word is evaluated by computing the difference in norm distance between the word from a masculine word group and a feminine word group. This approach can be easily extended to the text level by averaging the bias score of each word in text (Kwak et al., 2021) or taking the average of word vectors prior to bias evaluation.

## 3. Methodology

Using gender-indefinite words alone does not remove gender signaling completely, since agentic attributes (e.g., *active* and *adventurous*), are usually considered to be masculine, and communal attributes (e.g., *considerate* and *sympathetic*), are often considered feminine. These attributes may be favored for certain job positions and it may not always be possible to find neutral alternatives to replace them. Thus, it is more reasonable for the writer to keep these words while using words in the opposite gender to achieve inclusivity of both female and male applicants. Therefore, our methodology of mitigating bias in text involves the following steps:

1. Build an evaluation model of gender bias in words and texts;
2. Model probability distribution for the word occurrence of each group;
3. Provide guidance on how many words from each group should be used to mitigate bias.

### 3.1. Quantifying Gender Bias by Words

To measure gender bias in job advertisements, we use a list of words that contain gendered psychological cues that may signal the employer's gender preferences for job candidates. Our word list builds on established inventories, i.e., Bem (1974) and Gaucher et al. (2011) inventories, which contain words that are well-established in the literature to signal implicit gender bias. Our word list also includes a further set of cues that form part of the BIAS word inventory identified from job advertisements using expert coding that have not been included in the Bem and Gaucher inventories. For a full list of words used in our analysis and detailed information on the latter list, please see Konnikov et al. (2021). Moreover, we assume that every word in the masculine and feminine groups has a different level of signaling, so the words are sub-grouped further, in this case into two subgroups for computational simplicity, where each group of words is split into strongly or weakly masculine (or feminine) sets. In our setup, we used the GloVe Pennington et al. (2014) word embedding to achieve the split.

We assume that the overall bias expressed from a piece of text is equal to the sum of the bias expressed from each word, and more importantly, the effect of masculine words can be canceled out by the usage of feminine words in suitable proportions. Let *Y*_*i*_ denote the bias score of the *i*-th job text and ***X***_*i*_ = (*X*_*i*, sm_, *X*_*i*, wm_, *X*_*i*, sf_, *X*_*i*, wf_) denote the number of occurrences of each word in the *i*-th job text aggregated according to the word groups, i.e., *X*_*i*, sf_ denote the total number of *strongly feminine* words appearing in the *i*-th job text. Let β_0_, **β** denote the model parameter, then.


$$\begin{array}{l} Y_{i}=\beta_{0}+\beta^{⊤}X_{i}. \end{array}$$

### 3.2. Gender Bias Score at the Text Level

To collect the data for response *Y*_*i*_ in a comprehensive manner, we combine two different metrics to measure the bias at the text level. The first approach is based on the method proposed by Gaucher et al. (2011), which measures the bias purely through word counts and produces a score in {−1, 0, 1} for feminine, neutral and masculine, respectively. Since a discrete bias score is not adequate for capturing the degree of bias in texts, we adopted a word counting approach but modified the metric to give a continuous output in [−1, 1]. The score is computed as follows. The sign of the score is determined as in Gaucher et al. (2011) where a negative value represents feminine bias and a positive value represents masculine bias. The magnitude of the score is computed using the following equation:


(1) $$\begin{array}{l} |S_{1}|=\max\left\{\frac{X_{\text{mas}}-X_{\text{fem}}}{X_{\text{mas}}},\frac{X_{\text{fem}}-X_{\text{mas}}}{X_{\text{fem}}}\right\}, \end{array}$$

in which case when *X*_mas_ = *X*_fem_ the measure will output 0.

**Algorithm 1:**
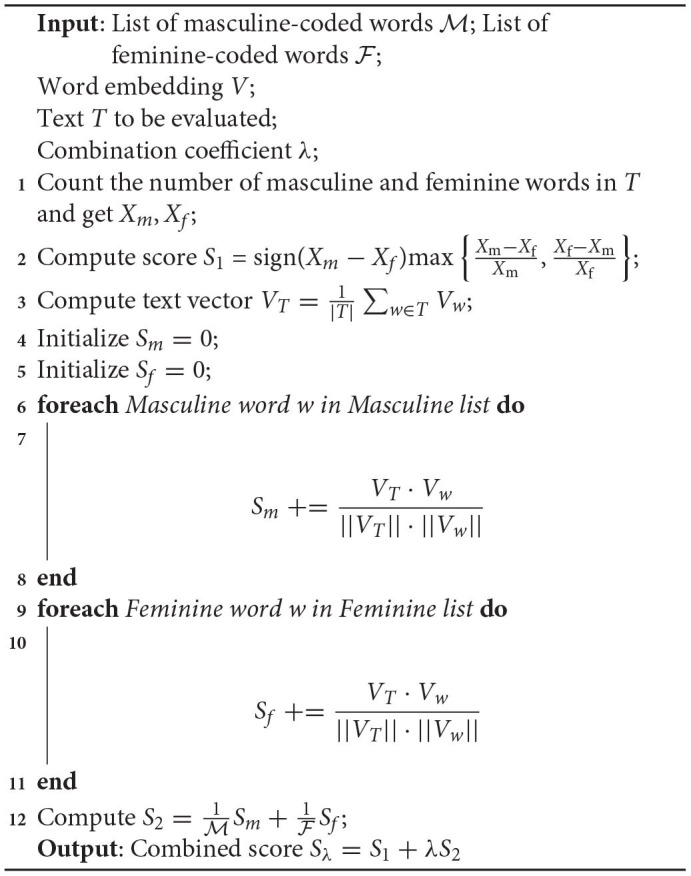
Text-bias evaluation

However, this measure does not consider potential differences in the levels of bias exhibited by different words. Thus, we consider a second bias metric similar to the Relative Norm Distance (RND) (Garg et al., 2018) or the Word Embedding Association Test (WEAT) (Caliskan et al., 2017). Since we need a text-level score, we average the word vectors from the same text to produce a text vector and compute its cosine distance to each of the masculine and feminine words in our word list. The difference in average cosine distance is our second score:


(2) $$\begin{array}{l} S_{2}=\frac{1}{\left|M\right|}\underset{w\in M}{\sum }\frac{V_{T}\cdot V_{w}}{\left||V_{T}||\cdot ||V_{w}|\right|}-\frac{1}{\left|F\right|}\underset{w\in F}{\sum }\frac{V_{T}\cdot V_{w}}{\left||V_{T}||\cdot ||V_{w}|\right|},\\ V_{T}=\frac{1}{\left|T\right|}\underset{w\in T}{\sum }V_{w}, \end{array}$$

where *T* denotes the text with its cardinality |*T*| defined as the number of words in *T*, *V*_*w*_ denote the word vector of word *w*, and M, F denotes the set of masculine and feminine words, respectively. The scores *S*_1_ and *S*_2_ are combined through a linear combination with coefficient λ to produce the final bias score for every text.

### 3.3. Bias Compensation

The combined scores can be used to estimate the model parameters $\left({\hat{\beta }}_{0},\hat{\beta }\right)$ through linear regression. With the model parameters $\left({\hat{\beta }}_{0},\hat{\beta }\right)$ estimated, the goal is to minimize the overall bias by adjusting the frequency of different word types *x*_*i*_. In theory, eliminating the use of gender-biased words may eliminate the bias completely. However, this is usually not possible since it can be hard to find neutral replacements for every word. Thus, we would like to seek a minimal adjustment to the word counts while reducing the bias. We would need to statistically model the word counts so that the debiased word count is highly correlated with the original word counts while satisfying some constraint (of zero bias) at the same time.

Although word counts are always integers, due to the complexity of solving probabilistic integer programming problems, we instead consider the continuous version with a deterministic objective:


(3) $$\begin{array}{l} {\hat{\beta }}_{0}+{\hat{\beta }}^{⊤}X_{i}=0. \end{array}$$

where ***X***_*i*_ is allowed to be a real vector which we can later round to an integer vector after debiasing.

With respect to the constraint above, the distribution of ***X***_*i*_ should also be modeled in order for the adjusted word counts to be as close to the original as possible. In this case, we consider the Gamma distribution as a continuous substitute for Poisson distribution. We assume that each job text is an instance of its own text distribution and thus every word count is from the same distribution but with distinct parameters, even for word counts of the same group. Therefore, rather than finding a common posterior distribution for the word count for each group, we would like to parameterize each distribution separately. To avoid over-complication, we leave 1 degree of freedom for each word count distribution to adjust its mean while using a common rate parameter for each group. Let ***X***_*i*_ = (*X*_*i*,sm_, *X*_*i*,wm_, *X*_*i*,sf_, *X*_*i*,wf_) and for each word group g∈G:={sm,wm,sf,wf}, *X*_*i,g*_ ~ Γ (α_*i,g*_, ψ_*g*_) with the density function given by


(4) $$\begin{array}{l} f_{i,g}\left(x\right)=\frac{\psi_{g}^{\alpha_{i,g}}}{\Gamma \left(\alpha_{i,g}\right)}x^{\alpha_{i,g}-1}\exp\left(-\psi_{g}x\right),\;\alpha_{i,g}:={\tilde{X}}_{i,g}\psi_{g}, \end{array}$$

where ψ_*g*_ is the fitted rate parameter using the collected word counts for each word group *g* separately and the mean of the distribution is chosen as the unadjusted word count ${\tilde{X}}_{i,g}$ for group *g* in text *i*. Now we have the following constrained distribution for job post *i*:


(5) $$\begin{array}{l} f_{i}\left(X_{i}\right)=\underset{g\in G}{\prod }f_{i,g}\left(X_{i,g};\alpha_{i,g},\psi_{g}\right)\;\text{w.r.t.}\;{\hat{\beta }}^{⊤}X_{i}=-{\hat{\beta }}_{0}. \end{array}$$

Finally, we can sample the unknown debiased word counts by simulating from the above distribution to give a natural choice of wording that also reduces the bias.

#### 3.3.1. Constrained Density Fusion

Let d=|G| denote the number of different word types. Recall that our target is to sample from the constrained product density function


(6) $$\begin{array}{l} f\left(X\right)\propto \underset{g\in G}{\prod }f\left(X_{g};\alpha_{g}\right)\;\text{w.r.t.}\;{\hat{\beta }}^{⊤}X=-{\hat{\beta }}_{0}, \end{array}$$

where ***X*** = (*X*_sm_, *X*_wm_, *X*_sf_, *X*_wf_).

Recently, the Monte Carlo Fusion algorithm (Dai et al., 2019) has been proposed to draw samples from product distributions similar to what we have in Equation (6) but without the constraint. Although the method cannot be directly applied, we note that the proposal of the algorithm is Gaussian in the target random variable. Since the constraint is linear, we can leverage the fact that a linearly constrained Gaussian distribution is still Gaussian to adapt the algorithm to our problem. Consider the following proposal distribution *h*(***X,Y***):


(7) $$\begin{array}{l} h\left(X,Y\right)\propto \overset{d}{\underset{j=1}{\prod }}f\left(X_{j};\alpha_{j}\right)\times \eta_{\hat{\beta }}\left(X\right)\times \frac{N\left(Y;X,TI_{d}\right){𝟙}_{\left\{{\hat{\beta }}^{⊤}Y=-{\hat{\beta }}_{0}\right\}}}{\eta_{\hat{\beta }}\left(X\right)}\\ \;\times Q, \end{array}$$

where


(8) $$\begin{array}{l} Q={𝔼}_{𝕎}\left[\Phi \left(W\right)\right],\;\Phi \left(W\right)=\exp\left[-\overset{d}{\underset{j=1}{\sum }}\int_{0}^{T}\phi_{i}\left(W_{s}^{\left(i\right)}\right)\text{d}s\right], \end{array}$$

is the expectation over the measure of Brownian bridges ***W*** of length *T* connecting ***X*** and ***Y***. Using ′ to denote the derivative symbol, the definition of ϕ_*i*_ is given by


(9) $$\begin{array}{l} \phi_{i}\left(x\right)=\frac{1}{2}\left[A_{i}^{\prime }{\left(x\right)}^{2}+A_{i}^{\prime \prime }\left(x\right)\right]-l_{i},\;A_{i}\left(x\right):=\log f_{i}\left(x\right), \end{array}$$

with *l*_*i*_ > −∞ being a lower bound of ϕ_*i*_. Finally


$$\begin{array}{l} \eta_{\hat{\beta }}\left(X\right)=\exp\left[-\frac{1}{2TB}{\left({\hat{\beta }}_{0}+{\hat{\beta }}^{⊤}X\right)}^{2}\right],\;B=||\hat{\beta }|{|}^{2}. \end{array}$$

Here the proposal distribution simulates a biased multidimensional Brownian bridge with the starting point following the joint product distribution $\prod_{j=1}^{d}f\left(X_{j};\alpha_{j}\right)$, which is the unconstrained target distribution, and its dimensions coalesce at time *T*, i.e., coordinates in each dimension at time *T* are the same. The simulation of coalescence is controlled by $N\left(Y;X,TI_{d}\right){𝟙}_{\left\{{\hat{\beta }}^{⊤}Y=-{\hat{\beta }}_{0}\right\}}$ which is normalized by the $\eta_{\hat{\beta }}\left(X\right)$. Finally, the correction *Q* is applied so that the marginal distribution of *Y* follows the target distribution. As *Q* cannot be directly evaluated, an event with probability *Q* is usually simulated to implement the correction. In this paper, we introduce an approximated approach to compute *Q* in the next subsection.

According to Dai et al. (2019), the marginal distribution of ***Y*** from Equation (7) without the constraint follows the unconstrained target distribution (Equation 6). Note that the distribution in Equation (7) has a dependency structure of three components, ***X***, ***Y***|***X*** and the diffusion bridge given ***X*** and ***Y***. Since the constraint only restricts the endpoints ***Y***, and the correction coefficient *Q* does not depend on the distribution of ***Y***, the unconstrained result can also be applied to our constrained case given that the constrained endpoint distribution can be defined. Clearly, with a linear constraint, we can find a natural definition for the constrained distribution of the endpoints ***Y***.

Since $\eta_{\hat{\beta }}$ cancels the residue function dependent on ***X*** from the integral of $N\left(Y;X,TI_{d}\right){𝟙}_{\left\{{\hat{\beta }}^{⊤}Y=-{\hat{\beta }}_{0}\right\}}$ with respect to ***Y*** over the constraint, sampling from the proposal density (Equation 7) can be done through the following steps:

1. Sample *X*_*j*_ ~ *f*(*X*_*j*_; α_*j*_), *j* = 1, …, *d*;
2. Sample $Y\sim N\left(X,TI_{d}\right){𝟙}_{\left\{{\hat{\beta }}^{⊤}Y=-{\hat{\beta }}_{0}\right\}}$;
3. First rejection step with probability $\eta_{\hat{\beta }}\left(X\right)\leq 1$;
4. Second rejection step with probability *Q*.

The last step can be done by simulating the event with probability equal to a one-sample estimate of *Q* (Beskos et al., 2006, 2008; Dai, 2017; Dai et al., 2019) and then accepting the sample with probability $\eta_{\hat{\beta }}\left(X\right)\leq 1$.

#### 3.3.2. Estimate Importance Weight

Recall that computing a one-point MC estimator of *Q* involves calculating an integral of stochastic process, which is generally intractable. Although it is possible to simulate an event of probability Φ(***W***), the rejection step could make the sampling inefficient. Instead, we may further estimate Φ(***W***) by constructing an unbiased estimator (Beskos et al., 2006; Fearnhead et al., 2008):


(10) $$\begin{array}{l} \hat{\Phi }=\overset{d}{\underset{i=1}{\prod }}\left\{e^{\left(\lambda_{i}-c_{i}\right)T}\lambda_{i}^{\kappa_{i}}\overset{\kappa_{i}}{\underset{j=1}{\prod }}\left[c_{i}-\phi_{i}\left(W_{s_{i,j}}^{\left(i\right)}\right)\right]\right\}, \end{array}$$

where λ_*i*_, *c*_*i*_ > 0 are parameters to be chosen and κ_*i*_ ~ Poi(λ_*i*_*T*), $s_{i,j}\sim U\left[0,T\right]$. Here *c*_*i*_ and λ_*i*_ are usually chosen as the upper-bound for the function ϕ_*i*_(*x*) and the upper-bound for *c*_*i*_−ϕ_*i*_(*x*), respectively, i.e., $\lambda_{i}=c_{i}-\underset{x}{\inf}\phi_{i}\left(x\right)$. Although the functions ϕ_*i*_ do not usually have a finite upper bound, it is possible to sample a compact interval for which the Brownian bridge *W*^(*i*)^ lives in and then compute the upper-bound for ϕ_*i*_. For the full implementation detail, please refer to Fearnhead et al. (2008).

By estimating the rejection probability, the rejection sampling can be turned into an importance sampling approach as presented in Algorithm 2. The shape parameters ψ_*g*_ in the algorithm are assumed to be known. In practice, we can estimate a shape parameter for each word group by fitting a Gamma distribution to the existing data. After simulating enough weighted samples, one can use the estimated mean as the debiased result. The rounded figure suggests how many words of each group should be included in the paraphrased text.

## 4. Application

In this section, we test the evaluation and debiasing strategy and algorithms on a real job post dataset that consists of 100,000 data points. The raw dataset contains job post information including *job title, job sector, job description, job location, full time or part time job*, and *salary*. Although job titles can be biased toward a certain gender, such gendered words have always appeared as part of a pair in the job titles in our dataset, e.g., postman/postwoman. Since the other fields are not the primary interest of this paper, we focused only on the *job description* data containing the main advertisement text.

**Algorithm 2:**
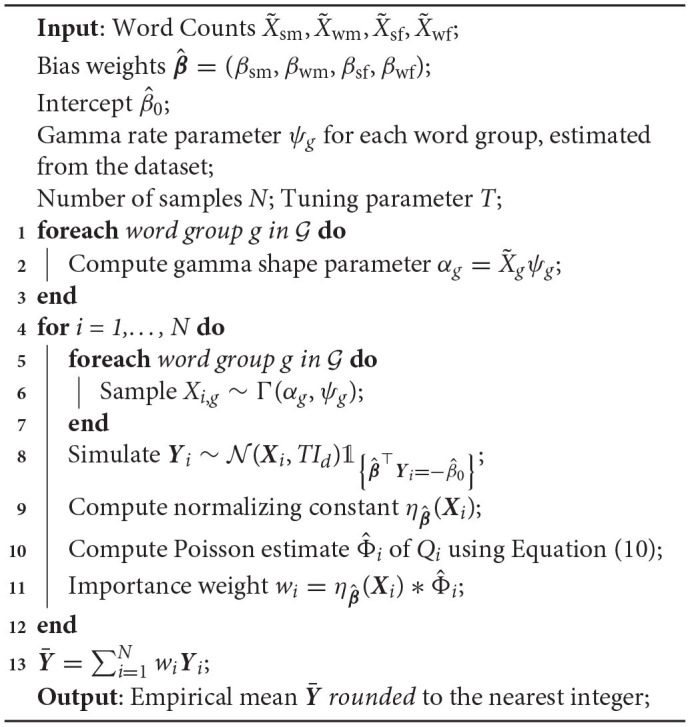
Bias reduction on word counts

The job texts are parsed from HTML to plain text and further processed to remove symbols. Then, the word counts are conducted by counting the total number of words in an advertisement and counting the occurrences of every word in our word list [see Konnikov et al. (2021) for a full list of words]. Some entries in the word list are root words, e.g., aggress*, in which case any variant that matches this root, e.g., *aggressive* and *aggression*, shares the same counter. Sometimes *regex* can match words that are misspelled, which should not be counted. In this case, we filter out these words by checking if they are contained in a dictionary. We used WordNet in our implementation.

In the end, the word counts are aggregated according to their word groups, {*strongly, weakly*} × {*masculine, feminine*}. The split is achieved using the GloVe word embedding (Pennington et al., 2014) by ranking the cosine similarity between each word and the gender direction *he*−*she*.

### 4.1. Bias Score

The text-level bias score is evaluated by combining two distinct measures based on word counts (Gaucher et al., 2011) and word embeddings (Garg et al., 2018), respectively, as described in Algorithm 1. Let *S*_λ_ denote the combined score using coefficient λ, in this case λ = 2 which gives the best regression outcome.

We formulate and solve the linear regression problem


$$S_{i,\lambda }=\beta_{0}+\beta_{\text{sm}}{\tilde{X}}_{i,\text{sm}}+\beta_{\text{wm}}{\tilde{X}}_{i,\text{wm}}+\beta_{\text{sf}}{\tilde{X}}_{i,\text{sf}}+\beta_{\text{wf}}{\tilde{X}}_{i,\text{wf}}+\epsilon_{i},$$

where ϵ_*i*_ is i.i.d. Gaussian noise and ${\tilde{X}}_{i,g}$ is the word count for word group *g* in the *i*-th text. The fitted parameters are shown in Table 1. We can see from the *R*^2^ that the regression model fits the estimated bias score reasonably well given the relatively simple and crude split of word groups. Let *S*_β_ denote the bias score estimated using the model parameters. Our fitted bias evaluation *S*_β_ is consistent with the combined bias score *S*_λ_ with a high Pearson's correlation, cor(*S*_λ_, *S*_β_) = **0.68**.

**Table 1:** Estimated weight for each word group.

	**Estimate**	**Std. Error**	***t* value**
Intercept	−0.1439***	0.0035	−40.78
Strong masculine	0.1580***	0.0008	199.42
Weak masculine	0.0073***	0.0004	16.39
Strong feminine	−0.1824***	0.0016	−115.45
Weak feminine	−0.1440***	0.0008	−175.35
*R* ^2^	0.465		

***p < 0.001.

The direction of bias in the bias score is recovered with *positive* toward *masculine* and **negative** toward **feminine**. In addition, the regression parameter validates the strong/weak split as the strong groups have coefficients with a larger magnitude than the weak groups. Overall, we can see that masculine words are assigned smaller weights, which can be caused by the wider usage of masculine words in the job text, similarly for the intercept which is negative.

### 4.2. Debiasing

With the bias weights $\hat{\beta }$ and intercept ${\hat{\beta }}_{0}$ estimated, we progress to sample the debiased word counts to reduce overall bias while keeping the relevant word counts close to the original version. For each word group, we fit a Gamma distribution to the 100,000 data points to get the corresponding rate parameter, (ψ_sm_, ψ_wm_, ψ_sf_, ψ_wf_) = (0.362, 0.258, 0.353, 0.350). Then we assume that the word count of group *g* in the *i*-th text *X*_*i, g*_, g∈G is a random variable that follows a Gamma distribution, $X_{i,g}\sim \Gamma \left({\tilde{X}}_{i,g}\psi_{g},\psi_{g}\right)$. Let *f*(*X*_*i, g*_) given by Equation (4) denote its density function. To debias each job text, we consider sampling from the following constrained product distribution:


$$f\left(X_{i}\right)=\underset{g\in G}{\prod }f\left(X_{i,g}\right)\;\text{w.r.t}\;{\hat{\beta }}^{⊤}X_{i}=-{\hat{\beta }}_{0}.$$

**Figure 1:**
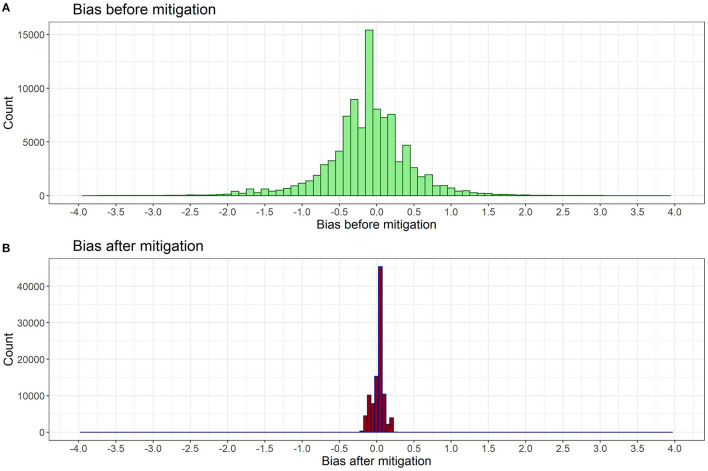
Histogram of bias score distribution **(A)** before and **(B**) after debiasing algorithm is applied. Both scores are measured using the fitted metric in Section 4.1.

The simulation is done by following Algorithm 2, and Figure 1 shows a comparison of bias score distribution before and after applying our bias mitigation approach. Before debiasing, the majority of job advertisements have bias scores between −2.0 and 2.0. After the bias mitigation, the bias score distribution is reduced to between −0.25 and 0.25 as shown in Figure 1B, with a high concentration around 0.

**Figure 2:**
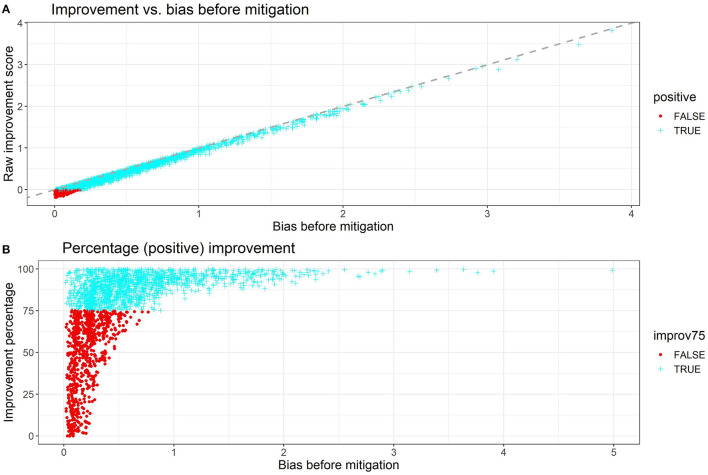
**(A)** Raw improvement and **(B)** percentage improvement plotted against the unsigned bias score before debiasing. In the percentage plot, only positive improvements are plotted since the points with negative improvement were already close to no bias and thus not relevant to the context.

The individual improvements are plotted in Figures 2A,B. The bias improvement is computed by taking the difference between the unsigned (absolute value) bias score before debiasing and the unsigned bias score after debiasing. To avoid overcrowding the scatter plot, both Figures 2A,B contain 3000 randomly sampled data points from the output. In Figure 2A, the bias improvement is strongly linear with the unsigned bias before debiasing and the linear relation has a slope close to 1. More importantly, the majority of points (**over 90%**) have positive improvements while the points with negative improvements have a very small unsigned bias score (<0.23) in the first place. In practice, the debiasing process of these points can be omitted since their original level of gender bias is close to 0.

Therefore, we only use the points with positive improvements in Figure 2B, where the percentage improvement is plotted against the unsigned bias score before debiasing. Overall, 67.7% of the points have percentage improvements greater than 75%, and the percentage increases to 99.9% for those with unsigned bias score greater than 0.75. From Table 2 we can see that the mean improvement gets better when we filter out texts with a lower magnitude of bias. For texts with a bias score of >0.75, the mean improvement percentage is 93.89% while the mean bias score after debiasing is 0.0677, which is very close to the mean debiased score across all data points 0.0628.

**Table 2:** Mean unsigned bias before and after debiasing with mean improvement and percentage improvement for different groups of data.

**Statistics**	**Among those with**			
	**All data**	**Improv. >0**	**Bias >0.23**	**Bias >0.75**
Mean |before|	0.4149	0.4536	0.6269	1.2362
Mean |after|	0.0628	0.0588	0.0647	0.0677
Mean improv.	0.3521	0.3948	0.5623	1.1685
Mean % improv.	32.77%	75.92%	86.08%	93.89%

## 5. Discussion

In this paper, we build a bias evaluation algorithm by grouping masculine and feminine words into strong and weak groups and assigning weights to each group to be used in the debiasing stage. We also introduce a debiasing strategy and algorithm by modeling the frequencies of each word group and sampling the word composition with less bias in our evaluation framework. We have shown that our bias weight is consistent with the grouping and that the debiasing algorithm is effective when dealing with texts of high bias scores. Although our test is based on reducing gender bias, our algorithm can also be applied in situations where the employer in a male-dominated industry may want to attract more female applicants by including more feminine words. This can be achieved by changing the constraint of zero bias to negative bias. In addition, although we used gender as a binary construct for illustrative purposes in this paper, our proposed algorithm can be extended to deal with multiple (linear) constraints. If the degree of bias toward and against a certain category can be measured, then our algorithms can reduce bias in that category axis by just imposing a constraint on the sampling algorithm.

Our algorithms also have a few limitations. First, we distinguish strong and weak words by computing the cosine similarity with the gender direction. This step may be refined by using human labeling and crowd-sourcing. It may also be attractive to weigh and model every word separately. However, this may incur high computational costs in the debiasing stage and would also require a larger corpus since not all target words appear in our dataset. Another limitation of our algorithm lies in its linear assumptions, as the sampling algorithm requires the model constraints to be linear. Thus, the feasibility of non-linear extensions to bias measurement may be limited. Finally, we are only able to suggest the word composition at the summary level since there is currently no suitable algorithm to expand our output back into a full text. Coordinated paraphrasing that controls the inclusion and exclusion of words in each sentence to achieve low bias may be possible, but it is overly complicated at the present stage, which should be a potential direction for future work.

## Data Availability Statement

The original contributions presented in the study are included in the article, further inquiries can be directed to the corresponding author.

## Author Contributions

AK, ND, YH, KH, LD, JA-A, MT, and SH: data curation. SH: formal analysis and writing—original draft. SH, HD, and JA-A: methodology. All authors conceptualization and writing—review editing.

## Funding

This work was supported by the Economic and Social Research Council (ESRC ES/T012382/1) and the Social Sciences and Humanities Research Council (SSHRC 2003-2019-0003) under the scheme of the Canada-UK Artificial Intelligence Initiative. The project title is BIAS: Responsible AI for Labour Market Equality.

## Conflict of Interest

The authors declare that the research was conducted in the absence of any commercial or financial relationships that could be construed as a potential conflict of interest. The handling editor declared a past collaboration with the authors JX and LK.

## Publisher's Note

All claims expressed in this article are solely those of the authors and do not necessarily represent those of their affiliated organizations, or those of the publisher, the editors and the reviewers. Any product that may be evaluated in this article, or claim that may be made by its manufacturer, is not guaranteed or endorsed by the publisher.

## Footnotes

^1^Whilst acknowledging gender as a non-binary construct, we operationalize gender as a dichotomized measure to refer to men and women for methodological and technical purposes in this paper.
